# Interactions Between Polyethyleneimine Xerogels and Acetic Acid Vapor from Degraded Cellulose Acetate. A Novel Therapy for Motion Picture Films Affected by the “Vinegar Syndrome”

**DOI:** 10.1002/marc.202500075

**Published:** 2025-04-22

**Authors:** Francesca Porpora, Luigi Dei, Camilla Forcellini, Carlotta D'Aleo, Lorenzo Lisi, Marianna De Sanctis, Emiliano Carretti

**Affiliations:** ^1^ Department of Chemistry “Ugo Schiff” University of Florence & CSGI Consortium via della Lastruccia, 3–13 Sesto Fiorentino Florence 50019 Italy; ^2^ Film Restoration Laboratory “L'Immagine Ritrovata” via Riva di Reno, 72 Bologna 40122 Italy; ^3^ National Research Council—National Institute of Optics (CNR‐INO) Largo E. Fermi 6 Florence 50125 Italy

**Keywords:** acidity, cultural heritage conservation, gas absorption, gels, motion picture films

## Abstract

Motion picture films made of cellulose acetate (CA) are subjected to degradation mainly due to the “vinegar syndrome”. The goal of this study is to investigate the absorption of acetic acid vapor by some polyethyleneimine (PEI) xerogels, aim at developing innovative, inexpensive, reusable, and easy‐to‐produce and handle chemical inhibitors for the “vinegar syndrome”. PEI‐based xerogels (SPEI) are obtained through cryo‐polymerization via epoxy‐amine reaction between PEI and 1,4‐Butanediol di‐glycidyl ether (BDDE). The intent is to enhance the absorption of the acetic acid vapor by the porous and high‐specific surface area network of the obtained PEI xerogel, allowing its neutralization through the free amino groups present in the gel. A chemical‐physical, morphological, and rheological characterization of the SPEIs is performed and their behavior in the absorption and desorption of acetic acid is also studied. The efficacy of the SPEI in inhibiting the “vinegar syndrome” is evaluated on both CA‐based real motion picture films, on which the deacetylation process is artificially and naturally induced. The characterization of degraded CA films stored with and without the inhibitor is evaluated using an already validated multi‐analytical protocol. The excellent results achieved open interesting perspectives for the conservation of these 20th‐century cultural heritage objects.

## Graphical abstract

Motion picture films made of cellulose acetate are subjected to chemical degradation primarily due to a deacetylation process known as *“vinegar syndrome”*. A polyethyleneimine‐based xerogel is proposed as an innovative chemical inhibitor to absorb and neutralize acetic acid by converting it into ammonium acetate through an acid/base reaction.

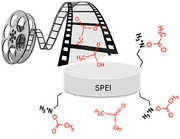

## 1 Introduction

The birth of cinema in the late 1800s and the evolution of the motion picture film industry in the following century are closely linked to the technological evolution of those years. From the 1920s the support of motion picture films was made of cellulose acetate (CA), which was chosen as a safer substitute for the more unstable and dangerous celluloid. Thanks to their lower flammability, motion picture films made of CA were called “safety films”. Unfortunately, this material also exhibited instability due to its characteristic degradation process, and during the 1960s and 1970s, more stable polyesters gradually replaced CA.^[^ ^1^, ^2^, ^3^, ^4^ ^]^ The most important degradation process that affects CA‐based media is traditionally called “vinegar syndrome”.^[^ ^4^ ^]^ This phenomenon concerns the cleavage of the ester bonds between the acetate group (CH_3_COO) and the cellulose backbone through ester hydrolysis (deacetylation), with the formation of hydroxyl groups and the release of acetic acid. This reaction is strictly influenced by temperature, moisture, and acidity: the released acetic acid acts as a catalyst for deacetylation inducing an autocatalytic process.^[^ ^3^, ^4^, ^5^, ^6^, ^7^, ^8^, ^9^ ^]^ Deacetylation and secondary reactions (e.g., depolymerization of the backbone^[^ ^4^ ^]^) lead to support alteration and severely compromise the films' usability and their future preservation. Therefore, it is essential to limit this phenomenon.^[^ ^10^ ^]^

Today, the most commonly used methods by conservators and archivists are based on two approaches: i) acting on the macro‐environment conditions (keeping the conservation conditions at both low temperature and Relative Humidity, RH) in relationship to the conservation status of the film^[^ ^7^, ^10^ ^]^; ii) subtracting the acetic acid from the degrading films in real‐time through its absorption and/or neutralization to avoid the trigger and the catalysis of the deacetylation process. Regarding the first approach (i), it only acts on the kinetics of the process,^[^ ^10^ ^]^ slowing down the rate of acetic acid production, and strictly limiting the use and the handle of the films. For the absorption and/or the neutralization of acetic acid, several systems have been proposed, (e.g., molecular sieves,^[^ ^5^ ^]^ activated charcoals,^[^ ^3^, ^11^ ^]^ CaCO_3_,^[^ ^11^, ^12^, ^13^, ^14^ ^]^ Na_2_CO_3_ and sodium polyacrylate^[^ ^15^ ^]^) and in each case there are some positive and negative aspects, as reported in the literature. The most recent proposal concerns the use of water‐stable Metal‐Organic Frameworks (MOFs),^[^ ^16^, ^17^, ^18^, ^19^ ^]^ which seems the best option, but has the main drawback of requiring complex and expensive syntheses.

On this basis, the goal of the present paper was to study the absorption of acetic acid vapor by some polyethyleneimine (PEI) xerogels aimed at developing innovative, inexpensive, reusable, and easy‐to‐produce and handle chemical inhibitors for the “vinegar syndrome”, able to prevent the deacetylation of CA films. The research strategy was based on the development and application of xerogels made of polyethyleneimine (SPEI). The aim was to obtain a porous structure via copolymerization by epoxy‐amine reaction between polyethyleneimine (PEI) and 1,4‐Butanediol di‐glycidyl ether (BDDE)^[^ ^20^, ^21^, ^22^ ^]^ and to use free amino groups of PEI to convert acetic acid into ammonium acetate through an acid/base reaction.^[^ ^23^ ^]^

## 2 Results and Discussion

**Figure 1:**
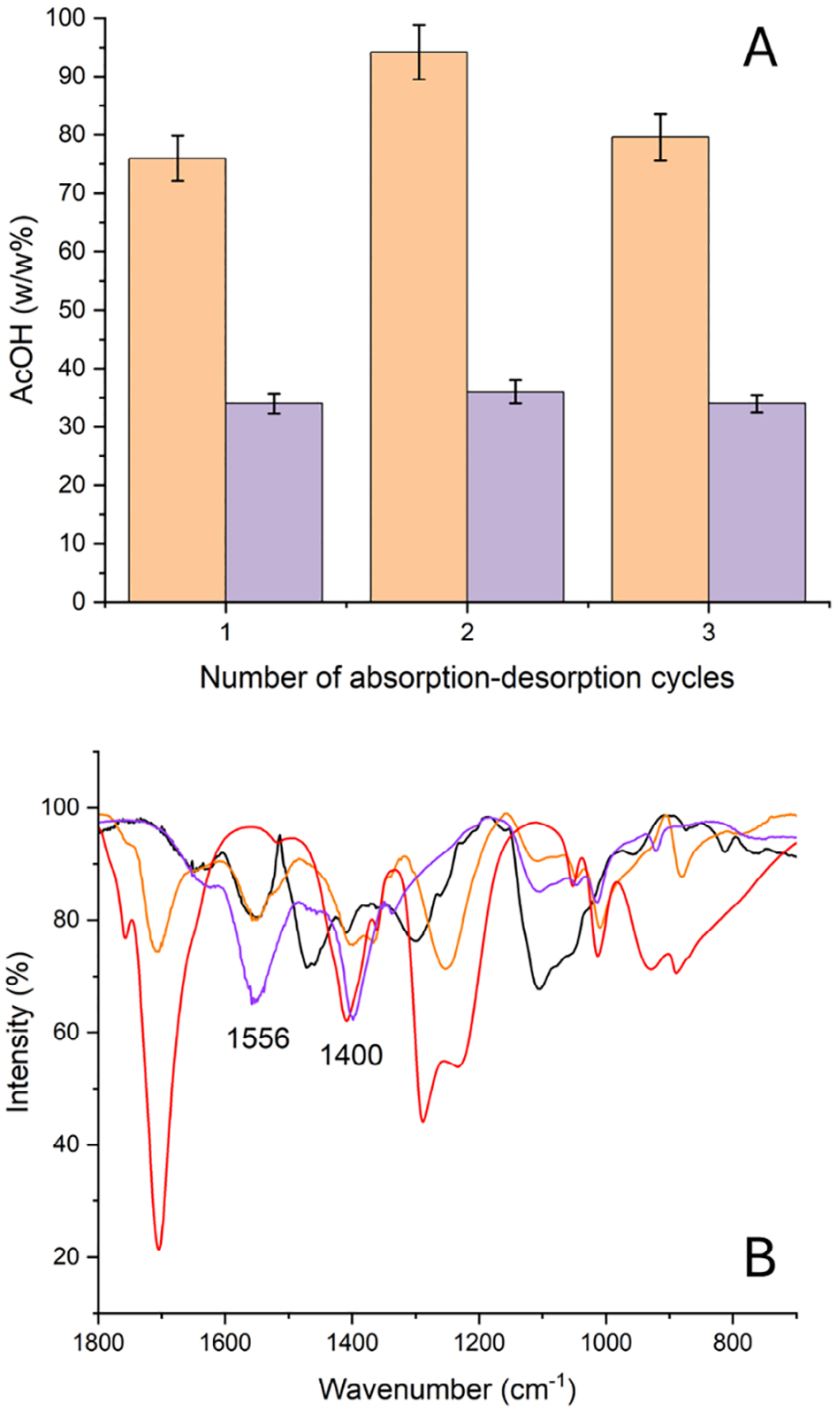
A) Amount of acetic acid (AcOH w/w%) absorbed inside a PEI xerogel with PEI:BDDE of 3:1 and a PEI concentration of 6 w/w% after three cycles of absorption‐desorption. In orange the amount of AcOH absorbed after 6 days of absorption test and in purple the AcOH absorbed after the desorption test (3 h at 15 mbar until constant weight is reached); B) FTIR‐ATR spectra of pure AcOH (red line), SPEI (black line), SPEI after the absorption of AcOH (orange line), SPEI after the desorption test (purple line).

Five xerogels were synthesized by varying PEI:BDDE ratio and PEI concentration to understand how changing these variables can influence the structure and performance of the system, mainly concerning acetic acid vapor absorption. Physico‐chemical characterization of the xerogels was conducted by multiple techniques: through Fourier Transform Infrared Spectroscopy (FTIR) and ThermoGravimetric Analysis (TGA), the spectroscopic and thermal properties were analyzed and the occurrence of the epoxy‐amine reaction was verified, even by comparing the results of Elemental Analysis (EA); through swelling tests in water and pH measurements, more information about the structure was obtained. Moreover, their morphology was investigated through Scanning Electron Microscopy (SEM) and X‐ray microtomography (micro‐TOM). Finally, their rheological behavior was studied, too. More details about syntheses routes and characterization data are reported in the Supporting Information file. In addition, fundamental tests to evaluate their capacity to absorb vapors from glacial acetic acid and their reusability were conducted: SPEI systems were shown to be able to absorb acetic acid from up to 75% of their weight at least for three cycles of acetic acid absorption (**Figure** **1**A). To verify the conversion of acetic acid into ammonium acetate, desorption tests were performed to remove the weakly bonded acetic acid until a constant weight was reached (≈35 w/w% of acetic acid absorbed, compared to the initial weight of the sponge, was completely converted into ammonium acetate, Figure 1A) and FTIR spectra were acquired after this procedure: the formation of ammonium acetate was detected by the presence of the two peaks at 1560 and 1400 cm^−1^ ascribable to the COO^−^ ion^[^ ^24^ ^]^ (Figure 1B).

Based on the above‐mentioned tests, the best SPEI system (with a PEI:BDDE ratio of 3:1 and a PEI concentration of 6%) was tested for inhibiting the “vinegar syndrome” on real motion picture films. For this purpose, a multi‐step method to artificially induce the deacetylation process in motion picture films through high‐acid catalysis was used. This method was already developed and tested in previous work^[^ ^25^ ^]^ and consists of two main steps: i) the deacetylation reaction is induced by exposing the film to an acid atmosphere; ii) RH and T were kept at 100% and 20 °C, respectively, to re‐start and further promote the evolution of the deacetylation reaction after applying the SPEI inhibitor at a distance of ≈0.5 cm from the motion picture film samples. Under these mild conditions, the chemical‐physical properties of the thermosensitive SPEI are preserved, allowing for the evaluation of their performance.

From the literature,^[^ ^26^ ^]^ it is known that the maximum concentration of acetic acid produced by a highly degraded motion picture film stored in a case is ≈20 ppm. Supposing that authors in ref.[26] consider a case for a motion picture film of ≈600 m (i.e. the biggest case commercially used with a diameter of ≈37 cm and height of 4.5 cm), the amount of acetic acid dispersed in the atmosphere is ≈100 mg. Taking into account that SPEI can absorb ≈75 w/w% of acetic acid (see Figure 1A), only 130 mg of the xerogel might be enough to absorb all the produced acetic acid. We decided to put 0.3 g of SPEI in each degradation chamber to optimize the performance of the system.

A scheme of the motion picture films subjected to the degradation protocol is reported in **Table** **1**.

**Table 1:** Resume of all the samples subjected to the artificial degradation protocol to evaluate the performance of SPEI in inhibiting the deacetylation process.

Sample	Duration of the first degradation step (days)	Duration of the second degradation step (days)	Treatment
P9_HCl5M	9	/	/
P12_ATM2.9_NT	9	12	/
P24_ATM2.9_NT	9	24	/
P36_ATM2.9_NT	9	36	/
P48_ATM2.9_NT	9	48	/
P12_ATM2.9_SPEI	9	12	SPEI4 xerogel at the bottom of the jar (0.3 g)
P24_ATM2.9_SPEI	9	24	
P36_ATM2.9_SPEI	9	36	
P48_ATM2.9_SPEI	9	48	

To monitor the evolution of deacetylation in motion picture films during the degradation protocol and evaluate the efficacy of the PEI xerogels as inhibitors, the variation in acetyl content (the amount of acetic acid esterified onto the cellulose backbone of the polymer),^[^ ^4^ ^]^ free acidity (non‐esterified acetic acid adsorbed on the film),^[^ ^27^ ^]^ and mechanical properties were evaluated through a multi‐analytical protocol.^[^ ^25^ ^]^ Acetyl content and free acidity are directly linked to deacetylation, which leads to depolymerization and induces changes in mechanical properties.^[^ ^1^, ^10^ ^]^ This approach allowed for the evaluation of the novel therapy's effectiveness against the “vinegar syndrome” by comparing the alteration in untreated and treated films subjected to the same artificial degradation protocol.

**Figure 2:**
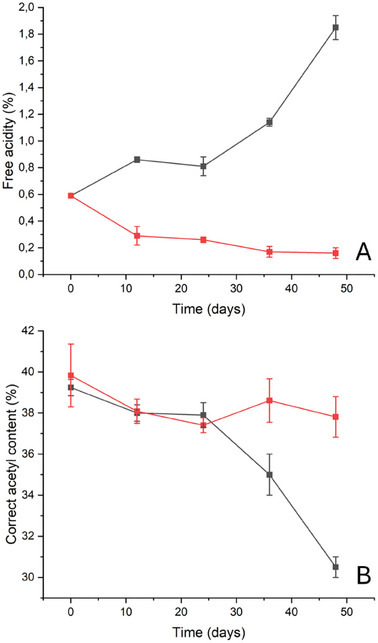
A) Free acidity (%) and B) correct acetyl content (%) calculated via the Heterogeneous Saponification Method (%) calculated for motion picture films subjected to the artificial degradation protocol with (red line) and without (black line) the SPEI inhibitor.

Regarding free acidity^[^ ^28^ ^]^(**Figure** **2**A), a significant decrease (≈50%) was observed in the first 12 days for samples stored with SPEI, likely due to the alkaline nature of the xerogel (this parameter decreased from 0.6 ± 0.1% for the sample P9_HCl5 M to 0.29 ± 0.07% for P12_ATM2.9_SPEI). Over the following 36 days, the rate of the process decreased, and the value registered for the P48_ATM2.9_SPEI sample was 0.16 ± 0.04%. An opposite trend was observed for all the samples degraded without SPEI: in this case, the free acidity value increased by almost 200% (from ≈0.6 ± 0.07% for the sample P9_HCl5 M to 1.85 ± 0.09% for the sample P48_ATM2.9_NT).

A similar trend was observed for the acetyl content (calculated via the Heterogeneous Saponification Method, HSM,^[^ ^28^ ^]^ Figure 2B), which remained nearly constant at 39% for the entire duration of the last step of the degradation protocol for the samples stored in the presence of SPEI systems. In contrast, for the samples degraded without inhibitors, a significant decrease in the acetyl content (≈20%) was observed after 24 days (from 39.2 ± 0.4% of the P9_HCl5 M sample to 30.5 ± 0.4% of the P48_ATM2.9_NT sample).

**Figure 3:**
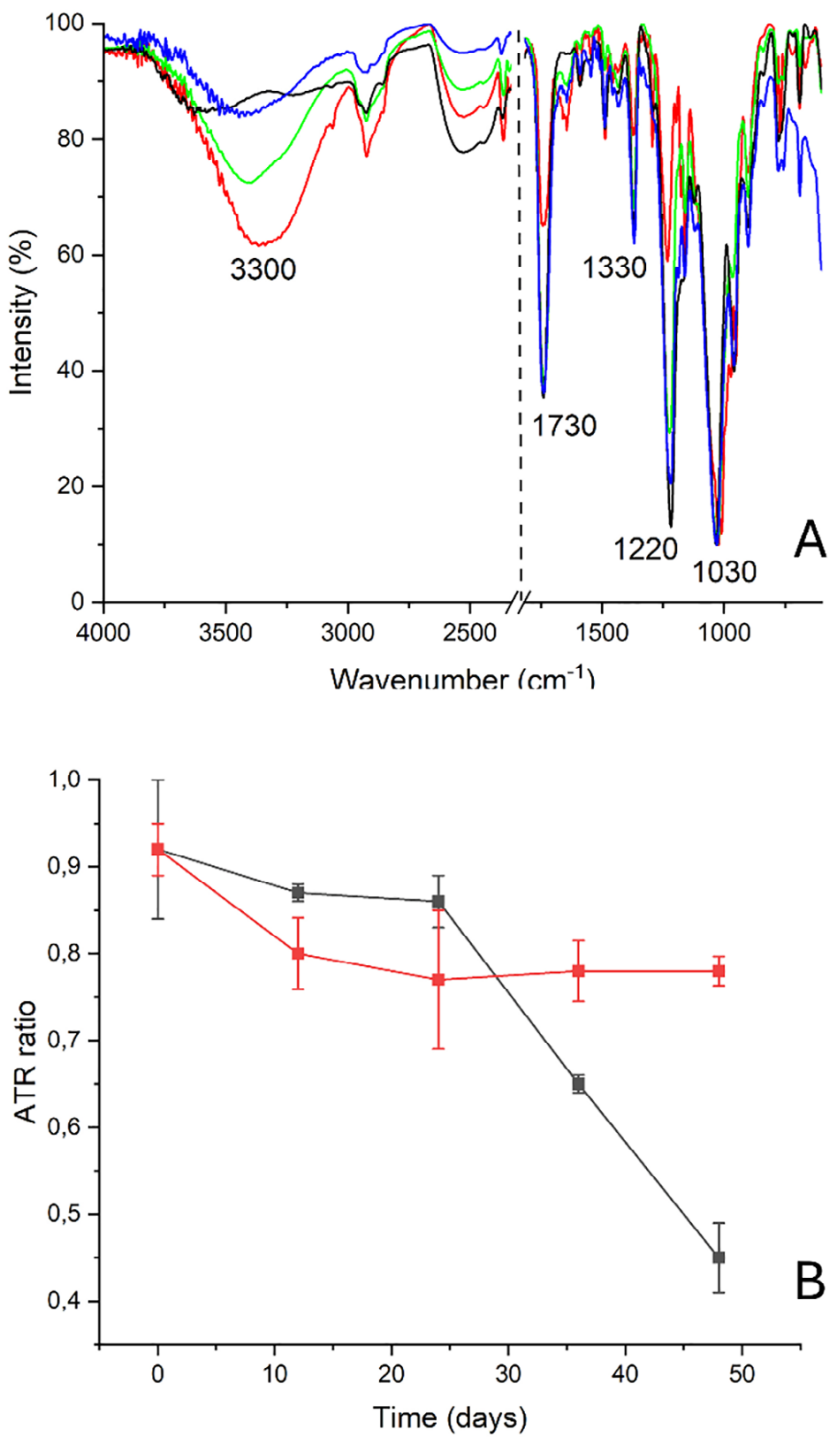
A) FTIR‐ATR spectra of P0 (black) and P9_HCl5 M (blue) and P48_ATM2.9_NT (red) and P48_ATM2.9_SPEI (green), in the range 4000–600 cm^−1^; B) FTIR‐ATR ratio I_1220_/I_1030_ for motion picture films degraded with (red line) and without (black line) the SPEI inhibitor.

Additionally, FTIR‐ATR spectroscopy^[^ ^19^, ^29^, ^30^, ^31^ ^]^ confirmed the effective performance of the SPEI systems. The spectra reported in **Figure** **3**A show that the peaks associated with the acetyl group (at 1220, 1330, and 1730 cm^−1^) in samples degraded in the presence of SPEI xerogels exhibited a slight decrease in intensity if compared to the P9_HCl5 M sample, but not as pronounced as in the samples degraded without any treatment. In contrast, films degraded without SPEI showed a significant reduction in the intensity of the acetyl group peaks. The peak associated with the hydroxyl group increased in intensity, but not to the same extent as in the samples degraded without the inhibitor. This increase could also be attributed to moisture absorption during the final phase of the degradation protocol, which involves storing the films at RH 100%.^[^ ^25^ ^]^ Concerning the ratio between the intensities of the peaks at 1220 and 1030 cm^−1^ (Figure 3B), for the samples degraded in the presence of SPEI, a modest decrease was observed after the 12th day (from 0.9 ± 0.05 for P9_HCl5 M to 0.80 ± 0.04 for P12_ATM2.9_SPEI) with subsequent stabilization from the 12th to the 48th day of the degradation process (the I_1220_/I_1030_ ratio was 0.78 ± 0.02 for P48_ATM2.9_SPEI). On the contrary, for films degraded in the absence of treatment, after the 24th day, the trend of this parameter confirmed the strong increase in the rate of the degradation process (the I_1220_/I_1030_ ratio was 0.45 ± 0.04% for P48_ATM2.9_NT).

**Figure 4:**
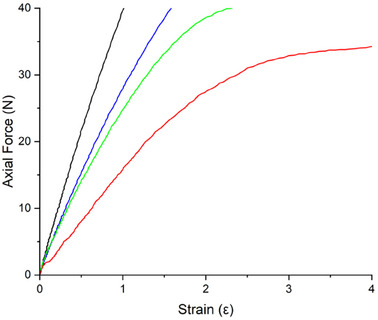
Axial Force *vs* Strain graph for P0 (black), P9_HCl5 M (blue), P48_ATM2.9_NT (red), and P48_ATM2.9_SPEI (green).

From tensile tests, the Young's Modulus (**Figure** **4**) calculated for the film treated with SPEI (P48_ATM2.9_SPEI: 11 ± 2 MPa) was lower than that measured for P9_HCl5 M (14 ± 1 MPa), but higher than that determined for the untreated sample (P48_ATM2.9_NT: 7.3 ± 1 MPa). This result further confirms the efficacy of the SPEI treatment.

The final step of this study concerned the evaluation of the performance of SPEI4 for inhibiting the “vinegar syndrome” on a film naturally affected by this disease.

The severe degradation status of PB_0 was assessed by comparing its free acidity, acetyl content, and ATR‐FTIR ratio with the ones of P0, a sample assumed to be in good conservation condition (**Table** **2**). The degraded motion picture films were placed in sealed aluminum bags and stored with (PB_SPEI_7 M) and without (PB_NT_7 M) SPEI at room temperature for 7 months.

**Table 2:** Resume of the results associated with real motion picture films naturally affected by the “vinegar syndrome” and stored with and without SPEI.

Sample	Free acidity [%]	Correct Acetyl Content [%]	ATR‐FTIR Ratio I_1220_/I_1030_
P0	0.02 ± 0.01	41.7 ± 0.7	0.91 ± 0.04
PB_0	0.25 ± 0.10	11.2 ± 0.5	0.30 ± 0.10
PB_NT_7M	0.30 ± 0.10	8.35 ± 0.7	0.16 ± 0.08
PB_SPEI_7M	0.10 ± 0.10	10.7 ± 0.7	0.40 ± 0.10

**Figure 5:**
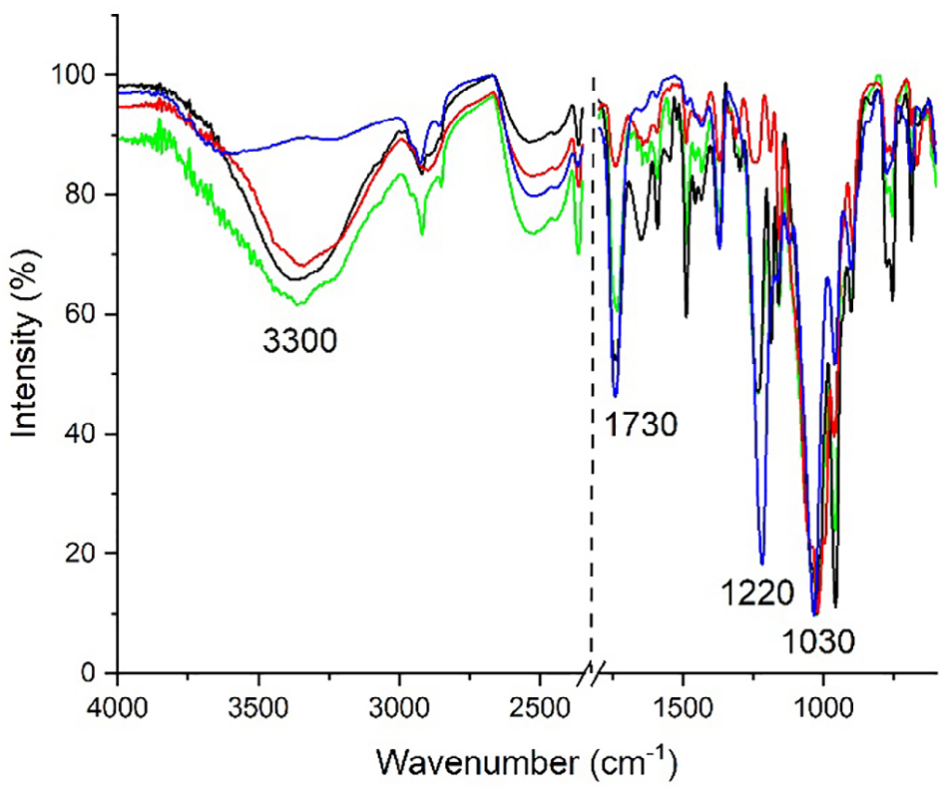
FTIR‐ATR spectra were acquired in the range 600–4000 cm‐1 for P0 (blue), PBO_NT (black), PB_NT_7 M (red), and PB_SPEI_7 M (green).

Comparing both the samples stored in an aluminum bag for seven months with (PB_SPEI_7 M) and without the inhibitor (PB_NT_7 M) to the sample at time 0 (PB_0), it was observed that, for the untreated sample, the free acidity remained almost unchanged compared to the initial value, while, for the treated sample, a decrease in the free acidity of ≈60% was observed (free acidity of PB_0: 0.25 ± 0.10%; PB_NT_7M: 0.30 ± 0.10%; PB_SPEI_7M: 0.10 ± 0.10%, Table 2). Additionally, the acetyl content of the films stored with or without the inhibitor differed: while the acetyl content of PB_SPEI_7 M remained almost the same as that of the sample at the beginning of the test (PB_0), for PB_NT_7 M a significant decrease of 25% was observed (Table 2). From ATR‐FTIR spectra, the difference between PB_NT_7 M and PB_SPEI_7 M samples at the end of the test was evident by examining the intensity of the peaks associated to the acetyl group (**Figure** **5**). While for PB_SPEI_7 M the intensity of the peaks at 1220, 1330 and 1730 cm^−1^ remained almost equal to those of PB_0, for PB_NT_7 M, the intensities of these peaks significantly decreased. Finally, the ratio between the intensities of the peaks at 1220 and at 1030 cm^−1^ was lower for PB_NT_7 M (0.16 ± 0.08) than for PB_ 0 (0.30 ± 0.10), while it remained almost unchanged for PB_SPEI_7M: (0.40 ± 0.10). Although the standard deviations were quite high (suggesting that deacetylation in real motion picture films occurred non‐homogeneously across the film surface), the FTIR‐ATR analysis corroborated the other data and indicated that SPEI was effective in inhibiting the progression of the deacetylation process in real motion picture films naturally affected by the “vinegar syndrome”.

## 3 Conclusion

In conclusion, xerogels synthesized through the epoxy‐amine reaction between PEI and BDDE and via cryo‐polymerization technique proved to be a highly effective approach for inhibiting the deacetylation process in real motion picture films. By adjusting the PEI:BDDE ratio and PEI concentration, it was possible to tailor the structure of the xerogels, thereby optimizing their mechanical properties and acetic acid absorption capacity. Furthermore, the ability to regenerate and reuse these xerogels provides a sustainable solution.

SPEI4 was tested for inhibiting the deacetylation in both artificially and naturally degraded real motion picture films: concerning the first case, while the deacetylation process advanced significantly in the absence of the xerogel, films stored with SPEI exhibited stabilization of both free acidity and acetyl content. The synergistic effects of acetic acid neutralization (due to the alkaline nature of the polymer) and moisture absorption played a crucial role in the effectiveness of the xerogel. Notably, this result was achieved even when the inhibitor was not in direct contact with the films, but placed ≈0.5 cm away, providing significant advantages in both usability and preservation of the optical properties of the films. Furthermore, the results highlighted that SPEI could be a promising solution also for inhibiting the degradation processes in real motion picture films naturally affected by the “vinegar syndrome.”

## Conflict of Interest

The authors declare no conflict of interest.

## Supporting information



Supporting Information

## Data Availability

Research data are not shared.
