# Supplementary material for: Interactions Between Polyethyleneimine Xerogels and Acetic Acid Vapor from Degraded Cellulose Acetate. A Novel Therapy for Motion Picture Films Affected by the “Vinegar Syndrome”
Source: Macromol Rapid Commun. 2025 Apr 22;46(14):2500075. doi: 10.1002/marc.202500075 (PMC12272524; doi:10.1002/marc.202500075)
Supplement: Supplementary file 1 — Supporting Information [file MARC-46-2500075-s001.docx]

Supporting Information

**Interactions between polyethyleneimine xerogels and acetic acid vapor from degraded cellulose acetate. A novel therapy for the treatment of motion picture films affected by the “vinegar syndrome”**

By: Francesca Porpora, Luigi Dei, Camilla Forcellini, Carlotta D’Aleo, Lorenzo Lisi, and Emiliano Carretti*

*Corresponding author

**Table of content**

**1. Experimental Section**

*1.1 Chemicals and materials*

*1.2 Synthesis of SPEI and Characterization*

*1.3 Evaluation of the performance of SPEI on real motion picture films*

**2. Supplementary results concerning SPEI syntheses**

*2.1 SPEI characterization*

**1. Experimental Section**

*1.1 Chemicals and materials*

Branched polyethyleneimine (PEI) with an average molecular weight of 750 kDa at 50% solution in water and 1,4-butanedioldyglycidyl ether (BDDE), cellulose triacetate (CTA, 43.3-43.9 wt.% acetyl content), cellulose diacetate (CDA, 39.8 wt.% acetyl content), and glacial acetic acid (AcOH, ≥ 99.7%), phenolphtalein were purchased from Sigma Aldrich. Sodium Hydroxide (≥ 98.5% pellets, anhydrous) was purchased by Acros Organics. Hydrochloric acid (37 wt%), and ethanol anhydrous denatured were purchased from Carlo Erba Reagents.

Water used for all the procedures was purified by a Millipore MilliQ Direct-Q® & Direct-Q UV water purification system (Water Resistivity: 18,2 MΩ at 25°C).

Products were used as purchased without further purification or treatment.

Real motion picture films used in this work came from two different sets.

- Frames subjected to artificial induction of the “vinegar syndrome” were from a black and white 16 mm reel called “*Vita di una Pianta*” (*Life of a Plant*, Fig. SI1A), an educational documentary of the “*Sezione Cinescolastica Paravia*” (*Movies for schools Paravia)*. These educational films were made in 1956 and distributed during the 1960s and the 1970s. The motion picture film (support and emulsion) was produced by Ferrania (Savona, Italy) and are made of cellulose acetate, as indicated by the typical edge code “SAFETY” ^[1]^, the polarization test ^[32]^, and the FTIR-ATR analysis presented in the main text (Fig. 1A). This reel was provided by the “Istituto Agrario ITAGR” (Florence, Italy).

- Some frames kindly gifted from the restoration laboratory “*L’Immagine Ritrovata*” (Bologna, Italy) were used to evaluate the performance of the proposed treatment on samples naturally affected by deacetylation (Fig. SI1B). It was a 35 mm color motion picture film that emitted a strong vinegar smell and appeared wavy and slightly deformed. The frames appeared yellowed, and the image showed signs of fading and shifts in color balance, likely due to spontaneous degradation processes affecting the dyes in the emulsion layer ^[1]^. Based these symptoms and the analysis reported below, it was reasonable to affirm that the film was affected by the “vinegar syndrome”.


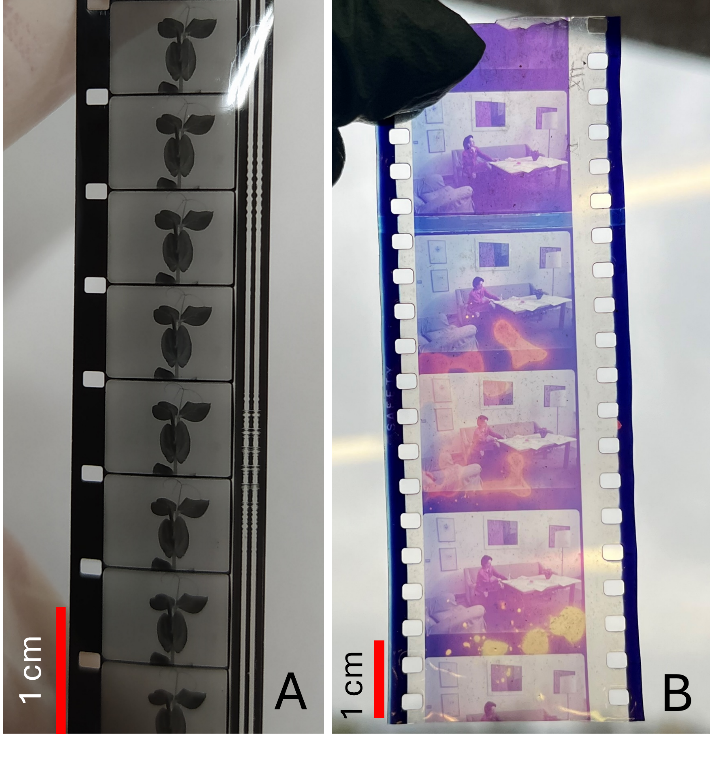


**Figure SI1.** (A) Frames from the motion picture films “Vita di una Pianta”; (B) Frames of a degraded cellulose acetate-based motion picture film.

*1.2 Synthesis of SPEI and Characterization*

Polyethyleneimine xerogels (sponges) (SPEI) were synthesized through cryo-polymerization via epoxy-amine reaction, using BDDE as a cross-linking agent ^[20–22]^. The synthesis was carried out by solubilizing PEI in water under stirring, followed by the addition of BDDE. The solution was vortexed for a few seconds, poured into a mold and stored at - 20°C for 24 hours. Afterward, the system was thawed and washed five times with water to remove unreacted reagents. Finally, the xerogel was dried at 50 °C. Several syntheses were performed, varying the concentration of PEI (4, 6, 8 w/w%) and the PEI:BDDE molar ratio (1:1, 2:1, 3:1), as reported in Table SI1.

| **Table SI1.** Scheme of the syntheses performed varying PEI concentration and PEI:BDDE molar ratio. |
| --- |
| \| Sample \| PEI concentration (wt%) \| PEI:BDDE \| \| --- \| --- \| --- \| \| SPEI 1 \| 4 \| 1:1 \| \| SPEI 2 \| 4 \| 2:1 \| \| SPEI 3 \| 4 \| 3:1 \| \| SPEI 4 \| 6 \| 3:1 \| \| SPEI 5 \| 8 \| 3:1 \| \|  \|  \|  \| |

*Fourier Transform Infrared Spectroscopy Measurements (FTIR-ATR).* FTIR spectra were collected using a Shimadzu, IRAffinity-1S Fourier Transform Infrared Spectrometer with the MIRacle Single Reflection Horizontal ATR accessory, equipped with a Diamond/ZnSe performance flat tip crystal plate. The resolution was set to 2 cm^-1^, with 64 scans and a range of 4000-600 cm^-1^. All the spectra were reported as Transmittance (%) versus wavenumber (cm^-1^).

*Thermogravimetric analysis (TGA).* Tests were performed in a nitrogen atmosphere at a heating rate of 10 °C/min over a temperature range of 25-500 °C, with an initial sample weight of approximately 5 mg using a SDT 650 thermal analyzer (TA).

*Elemental Analysis.* Quantitative elemental analysis on C and N was conducted using a Thermoscientific FlashSmart Elemental Analyzer CHNS/O (accuracy ± 0.1%), calibrated with cystine, methionine, sulfanilamide as standards. The percentage of each element was expressed as the weight percentage of the total sample. With these data, the C/N ratios for the unreacted PEI and all the xerogels were calculated.

*pH measurements.* pH measurements were carried out on the xerogels with a WTW™ ProfiLine™ pH 3310, calibrated with NIST/DIN standard pH 6,865 e pH 9,180. Samples were saturated with water and the the pH-meter probe was placed in contact with the samples for a few seconds. Five measurements were taken from different points on the surface of each sample. The average values and corresponding standard deviations are reported.

*Swelling tests.* Swelling tests were performed on xerogels in water and the kinetics of the absorption was monitored by measuring the weight increase as a function of time. The procedure involves drying the samples to their dry weight (W_dry_), then immersing them in 10 mL of water in a sealed vial. At defined time intervals, the samples were removed, gently and rapidly dabbed on a Whatman filter paper, and weighed (W_wet_) until an asymptotic value, corresponding to the saturation of the system. The Weight Increase (WI) in percent was calculated according to the following equation:

${SW\%}=\frac{W_{\mathrm{wet}}-W_{dry (time = 0)}}{W_{dry (time = 0)}}\cdot100\%$ (1)

The maximum amounts of the absorbed solvents (SW%) was determined from the asymptotic values of the curves. The results were expressed as the average value and with the corresponding standard deviation, calculated from three replicate tests of the same system.

*Scanning Electron Microscopy (SEM).* SEM micrographs were collected using a Hitachi SU3800 instrument, operating in high vacuum mode with an acceleration potential of 15 kV. The samples were metallized with gold vapor under vacuum.

X‑ray microtomography (micro-TOM). X-ray microtomography measurements were carried out with a Skyscan 1172 high-resolution MicoCT system at CRIST Centre, University of Florence (Italy) on a sample of ~ 1x0.5x0.25 cm. The X-rays tube equipped with a tungsten anode was operated at 100 kV and 100 μA. Placing the sample between the X-ray source and the CCD detector, 2D X-ray images were captured over a 180-degree rotating sample with a slice-to-slice rotation angle of 0.3. The spatial resolution of the image was kept in a range of 4 μm in terms of pixel size. The 3D image was reconstructed from the projections using the Nrecon software (Bruker μ-CT 1.6.10.2). After reconstruction, the image was analyzed to obtain information on the xerogel structure, such as the pore size distribution, through the CTAnalyser software (Bruker μ-CT 1.18.8.0). A 3D representation in false-colour was realized by the CTVox software (Bruker μ-CT 3.3.0).

*Rheology.* Rheology measurements were carried out on water-saturated samples using a TA Discovery HR-3 hybrid rheometer according to the following procedure. Frequency sweep measurements were performed to monitor the behavior of the elastic modulus (G') and the viscous modulus (G'') as a function of the oscillation frequency at constant oscillation amplitude. The range for this measurement was determined through amplitude sweep measurements of G' and G'' at a constant frequency sweep of 1 Hz, varying the oscillation amplitude. The normal force was set equal to 0.5 N for all the measurements.

*Acetic Acid Absorption-Desorption Tests.* To evaluate the capacity of the xerogels to absorb gaseous acetic acid, they were dried and weighed (W_dry_) and placed in sealed jars with vials containing 8 mL of glacial acetic for six days (until saturation). Then, the samples were left to equilibrate for six more days at controlled temperature and humidity (20 °C – RH 50%) and weighed (W_ab_).

The amount of absorbed acetic acid (AcOH_ab_%) was evaluated using the following equation:

$\mathrm{AcOH}_{\mathrm{ab}}\%=(\frac{W_{\mathrm{ab}}-W_{\mathrm{dry}}}{W_{\mathrm{dry}}})\cdot100$ (2)

Where W_dry_= weight of the dried sample and W_ab_= weight (g) of the sample after the absorption test. To further investigate the interaction between the xerogels and the acetic acid, desorption tests were performed ^[33]^. Samples previously subjected to absorption tests and equilibrated at room temperature for 6 days were maintained at low pressure (approximately 15 mbar) until a stable weight was achieved. The amount of strongly bound acetic acid (AcOH_sb_%) was calculated using the following equation:

$\mathrm{AcOH}_{\mathrm{sb}}\%=(\frac{W_{\mathrm{des}}-W_{\mathrm{dry}}}{W_{\mathrm{dry}}})\cdot100$ (3)

Where W_dry_= weight (g) of the dried sample, W_des_ = weight (g) of the sample after the desorption test.

The weakly-bonded acetic acid (AcOH_wb_%) after the equilibration period was calculated as:

$\mathrm{AcOH}_{\mathrm{wb}}\%={AcOH}_{ab}\%-{AcOH}_{sb}\%$ (4)

Three measurements were taken for each sample and the average values and corresponding standard deviation are reported. FTIR-ATR spectra were collected before and after the absorption tests and after the desorption test.

*Regeneration and reuse tests*. To regenerate the material for reuse, xerogels previously subjected to acetic acid absorption tests were immersed in a 1M NaOH solution for 10 minutes ^[34]^, then washed three times for 20 minutes with water and dried at 50 °C. To evaluate the possibility of reusing these systems, three cycles of absorption-regeneration were carried out. The performance of the xerogels after each absorption test was evaluated through gravimetric analysis, as described in the previous paragraph. Three measurements have been performed for each sample, and the average values with corresponding standard deviations are reported.

*1.3 Evaluation of the performance of SPEI on real motion picture films*

To evaluate the performance of the SPEI on motion picture films, two experiments were conducted.

The first experiment focused on assessing the capacity of the proposed treatment to inhibit the “vinegar syndrome” in motion picture films on which the deacetylation process had been artificially induced ^[25]^.

The second experiment aimed to evaluate the performance of the xerogels on samples that were naturally affected by the “vinegar syndrome”. The film was cut into two fragments, each measuring 10x3.5 cm and placed into two sealed metal-foil bags (15x20x0.22 cm) internally coated with a layer of polyethylene. A SPEI xerogel of the same dimension of the films was placed between the two fragments, in contact with the support sides. In parallel, an equal amount of film was put inside a bag without the treatment. The samples were stored for 7 months (210 days) at room temperature and humidity-controlled conditions (25 ± 3 °C and 50 ± 2%) inside the bags to promote the deacetylation process by increasing the acetic acid concentration in the enclosed environment. After this period, treated and untreated samples were compared to assess the potential effect of the inhibitor.

Methods and techniques used to monitor the evolution of the deacetylation process are detailed in a previously published paper ^[25]^.

For free acidity and acetyl content values, the reported results are the average values along with the corresponding standard deviations, which were calculated from three replicas of the same sample. For tensile tests, five replicas were analyzed. Regarding FTIR-ATR measurements, five spectra were collected from different areas of the same sample, and the average values along with standard deviations are reported.

**2. Supplementary results concerning SPEI syntheses**

*2.1 SPEI characterization*

Several syntheses of polyethyleneimine xerogels (SPEI) were carried out by varying the PEI:BDDE ratio and the PEI concentration.

*FTIR-ATR Spectroscopy.* The chemical composition of the polyethyleneimine xerogels was investigated by means of FTIR-ATR spectroscopy. In Fig. SI2, the spectra of the five SPEIs and that of unreacted brunched PEI are shown. The characteristics peaks of PEI ^[35,36]^ were visible in all the xerogels spectra: the out-of-plane wagging at 940 cm^-1^, the C-NH_2_ stretching at 1044 cm^-1^, the C-N-C stretching at 1110 cm^-1^, the C-H bending at 1463 cm^-1^, the NH bending of primary amine at 1600 cm^-1^, the C-H stretching at 2948 and 2845 cm^-1^ and the N-H stretching between 3100-3600 cm^-1^. When comparing the spectra of the xerogels to that of PEI, an increase in the intensity of the peak at 1105 cm^-1^ (ascribable to both the C-N-C and the C-O stretching) was appreciable. This trend can be attributed to the opening of the epoxy ring and the subsequent epoxy-amine reaction, leading to the formation of new C-N-C bonds ^[20,22]^. In all the SPEI spectra, two further peaks were observed at 1280 cm^-1^ and 1330 cm^-1^: the first peak can be assigned to the stretching of the C-O of the epoxy ring, which suggests the presence of unreacted epoxy rings on the BDDE molecules grafted to PEI; the second peak is likely associated with the O-H bending of the BDDE chain ^[20]^. In the xerogel spectra, a peak at 1555 cm^-1^ was present, likely attributed to the NH bending of secondary amines, and its intensity increased following the epoxy-amine reaction ^[37]^.

| 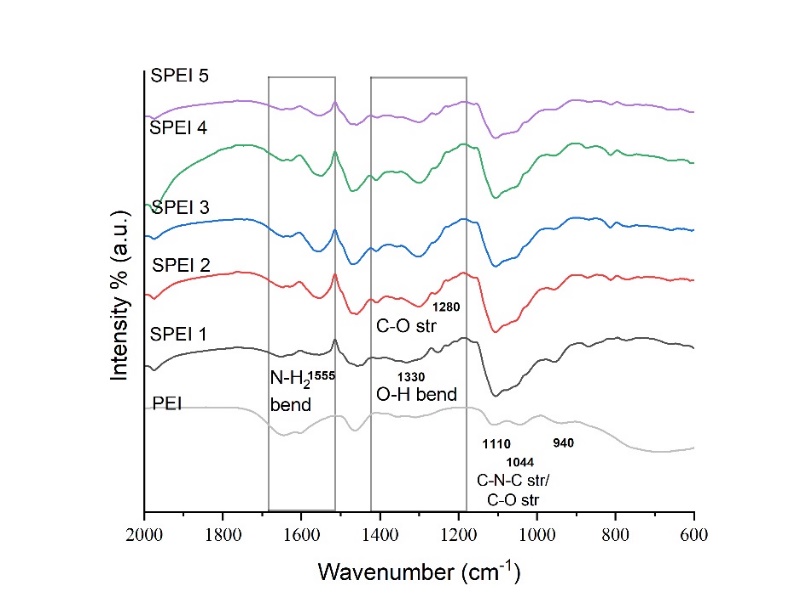 |
| --- |
| **Figure SI2.** FTIR-ATR spectra of unreacted brunched PEI (gray) and of SPEI 1 (black), SPEI 2 (red), SPEI 3 (blue), SPEI 4 (green), SPEI 5 (purple). |

*Thermogravimetric analysis.* In Fig. SI3, the TGA and DTG curves of pure PEI and of the SPEI4 system are presented. All the TGA and DTG curves of the other SPEI xerogels exhibited a similar trend to that of SPEI4, which is shown in Fig. SI3 as an example. Comparing the thermogravimetric profile of unreacted PEI and all the xerogels, a shift in the pyrolysis temperature ^[38]^ was observed (from 250-400 °C for PEI, to 290-390 °C for the xerogels). This shift indicates the success of the epoxy-amine reaction between the BDDE and PEI, leading to the formation of crosslinked network.

| 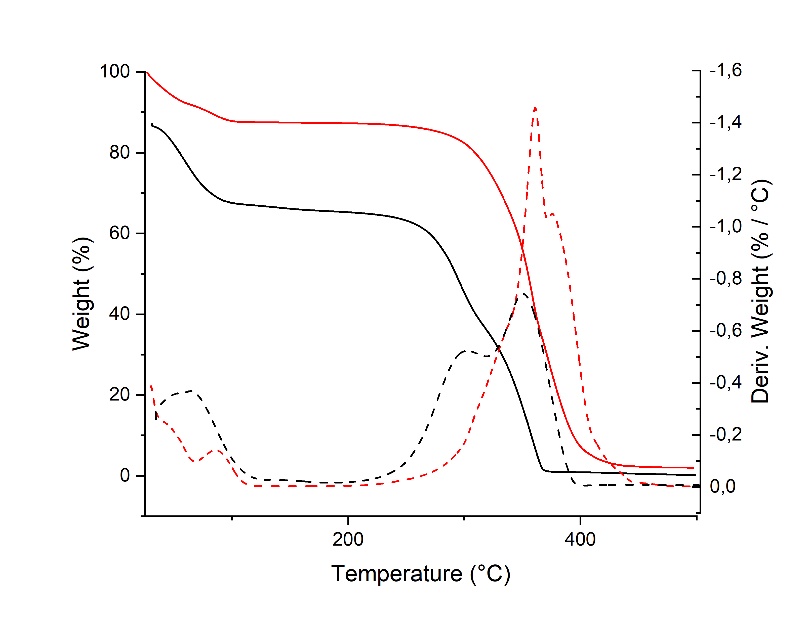 |
| --- |
| **Figure SI3**. TGA (solid line) and DTG (dashed line) curves of PEI (black) and SPEI 4 (red). |

*Elemental Analysis.* From the elemental analysis carried out on both unreacted PEI and xerogels, the C/N ratio was calculated, as shown in Fig. 4A-B: the C/N ratio of the unreacted PEI (1.8, the “0” point in Fig. SI4) was lower than that of all the xerogels (ranging from 4.5 to 3.4). This trend can be attributed to the BDDE molecules, which serves as a “source” of carbon atoms and are crosslinked and/or grafted to PEI. As expected, a decrease in the amount of BDDE added (while maintaining the same PEI concentration) resulted in a lower C/N ratio (4.5 for SPEI 1, 4.3 for SPEI 2 and 3.4 for SPEI 3, Fig. SI4A). On the other hand, increasing the PEI concentration while maintaining a constant PEI:BDDE ratio appeared not to affect the C/N ratio, which was 3.4 for SPEI 3, 3.65 for SPEI 4 and 3.4 for SPEI 5 (Fig. SI4B).

*Swelling tests.* Swelling tests (Fig. SI4C-D) in water were performed to obtain more information about the structure of the SPEI xerogels, with a comparison of the C/N results. A more detailed explanation of this point will be reported below. All samples exhibited the same swelling kinetics, reaching equilibrium 120 minutes after immersion in water. The maximum swelling in water (Eq. 1, Fig. SI4C-D) ranged from 771% to 1700%, confirming the high hydrophilic character of PEI-based systems.

As thr PEI:BDDE ratio increased, the total amount of adsorbed water also increased (775±9 w/w% for SPEI 1, 1046±9 w/w% for SPEI 2, 1700±10 w/w% for SPEI 3, Fig. SI4C). In contrast, increasing the PEI concentration led to a decrease in the total amount of absorbed water (1700±10 w/w% for SPEI 3, 1030±16 w/w% for SPEI 4, 771±9 w/w% for SPEI 5, Fig. SI4D). This decrease is likely due to the increased spatial density of the polymer network, which results in a reduced volume available for water adsorption.

As reported in the literature ^[39]^, comparing the swelling capacity of our systems with the C/N ratios can provide insights into the reaction process and the pathways involved during the syntheses. High swelling values associated with high C/N ratios suggest that the predominant reaction between PEI and BDDE was the grafting of the cross-linker as a side chain, rather than the formation of cross-links between PEI molecules via the reaction of the epoxy groups of the BDDE with the free -NH_2_ groups of PEI. By examinging the data of the xerogels obtained by increasing the PEI:BDDE ratio (SPEI 1, SPEI 2 and SPEI 3), we observed a decrease in the C/N ratio due to the decrease in BDDE (Fig. SI4A). On the contrary, an increase in the swelling capacity was reported (Fig. SI4C), probably due to the formation of a less structured network. Regarding the increase in PEI concentration, with a constant PEI:BDDE ratio (SPEI 3, SPEI 4 and SPEI 5), no significant variation in the C/N ratio was observed (Fig. SI4B). A decrease in the swelling capacity was observed as the PEI concentration increased (Fig. SI4D). This suggests that the grafting reaction was favored in less concentrated samples (SPEI 3 and 4), while crosslinking occurred more frequently in the more concentrated xerogels (SPEI 5).

pH measurements. pH measurements (Fig. SI4 E-F) were carried out to gain insight into the amount of free -NH_2_ groups, which determine the alkaline character of the xerogels, and to assess the capability of the xerogels to neutralize the acetic acid produced by motion picture films affected by the “vinegar syndrome”. The pH of pure PEI was about 12.5, while the pH of all the SPEI xerogels remained alkaline, ranging between 8 and 9. This reduction in pH provides further confirmation of the occurrence of the epoxy-amine reaction, which led to a decrease in the free -NH_2_ groups. Comparing the various systems, we observed that the pH data were consistent with the elemental analysis results: the pH increased with the PEI:BDDE ratio (pH 8.7±0.1 for SPEI 1, 9±0.1 for SPEI 2 and 9.5±0.1 for SPEI 3, Fig. SI4E), reflecting the lower extent of the reaction and a higher amount of free amino groups. Consistent with the trend of the C/N ratio, the pH appeared to be almost independent of PEI concentration when the PEI:BDDE ratio was maintained constant (pH 9.5±0.1 for SPEI 3, 9.7±0.1 for SPEI 4 and 9.4±0.2 for SPEI 5, Fig. SI4F).

| 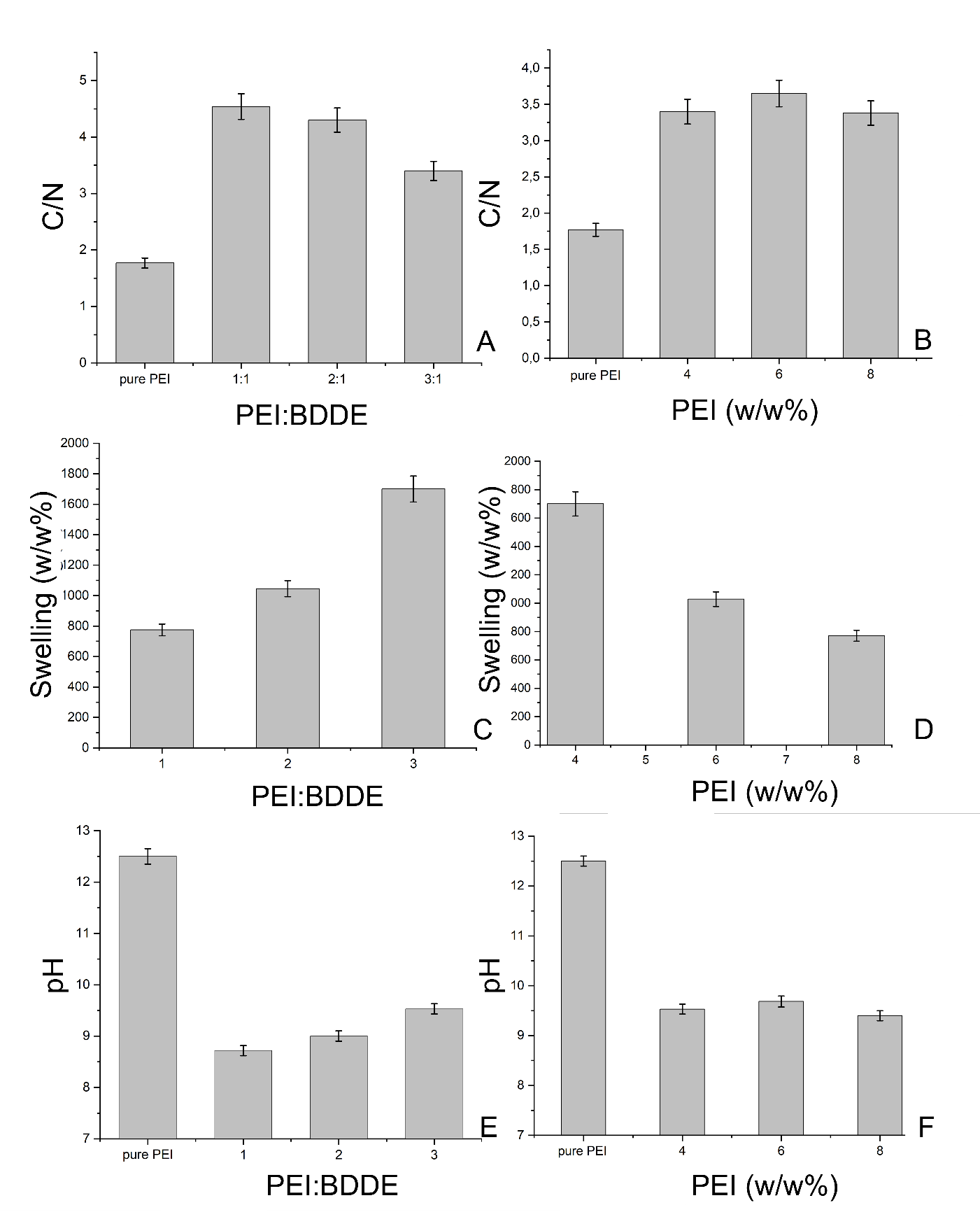 |
| --- |
| **Figure SI4.** C/N ratio vs PEI:BDDE ratio (A) and PEI concentration (w/w%) (B); swelling capacity *vs* PEI:BDDE ratio (C) and PEI concentration (w/w%) (D); pH data vs PEI:BDDE ratio (E) and PEI concentration (w/w%) (F). |

*Scanning Electron Microscopy.* Previous hypotheses were consistent with the information obtained from SEM analysis. The micrographs shown in Fig. SI5 indicate that as the PEI:BDDE ratio increased from 1:1 (SPEI 1, Fig. SI5A) to 1:3 (SPEI 3, Fig. SI5B), the porosity of the xerogel also increased. This behavior could be ascribed to a decrease in the spatial density of the crosslinking between PEI chains formed by BDDE molecules. On the other hand, when the PEI concentration was increased from 6% (SPEI 4, Fig. SI5C) to 8% (SPEI 5, Fig. SI5D), the structure of the xerogel became more compact and was characterized by the presence of lamellar patterns.

| 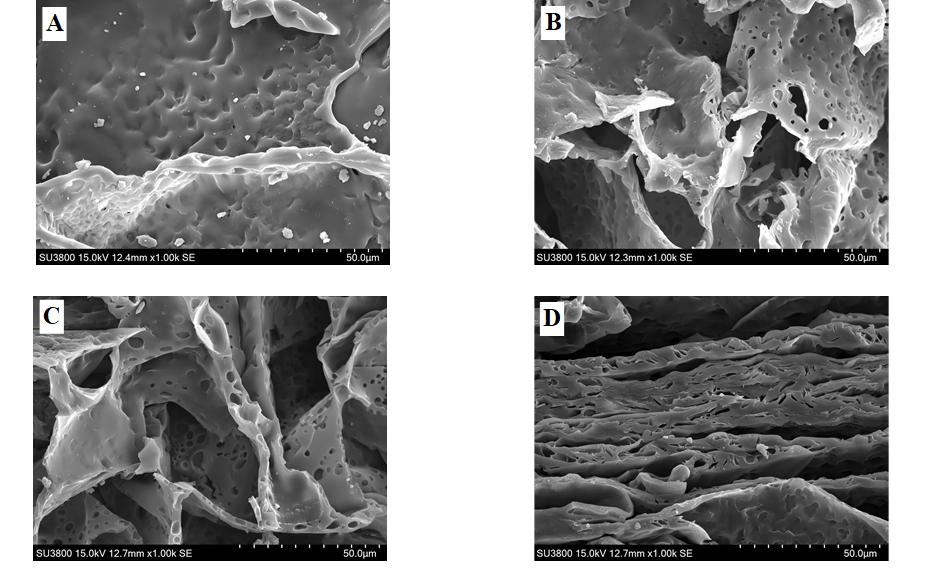 | |
| --- | --- |
| **Figure SI5.** SEM micrographs performed with 1.00kx magnitude of (A) SPEI 1, (B) SPEI 3 (C) SPEI 4 (D) SPEI 5. |  |

*X-ray micro-tomography.* For SPEI 4, a further investigation of the pore size distribution (Fig. SI6A and B) was carried out through µ-TOM on SPEI4. In terms of pore dimension, the micropore sizes range from 22 to 85 µm^3^, with a peak at 45 µm^3^. The total porosity percentage relative to the sample volume is 78.29%, with a higher percentage of open porosity compared to closed porosity (78.29% vs. 0.02%).

*
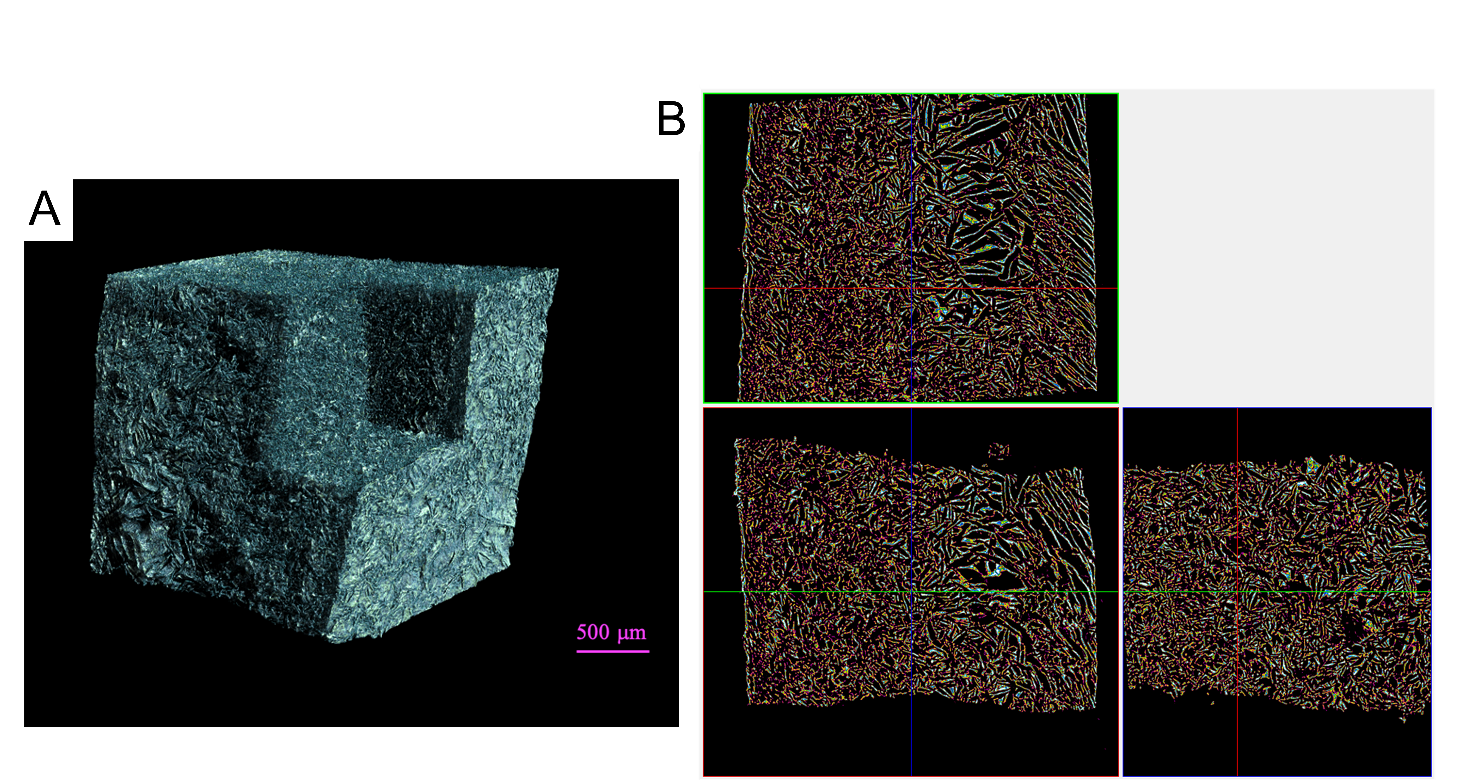
*

**Figure SI6.** (A) µ-TOM 3D reconstruction in false color of SPEI4 with sections (B).

*Rheology.* Oscillatory rheological measurements were performed to investigate the effects of the chemical composition of the systems on their mechanical properties and crosslinking density. Frequency sweeps (e.g., the behavior of the moduli G′ and G" as a function of the applied shear stress frequency) were conducted within the linear viscoelastic range, previously determined for each sample through amplitude sweep tests ^[40]^. All investigated samples (Fig. SI7A and B) exhibited a solid-like behavior typical of gels, as evinced by the elastic modulus G′ being consistently higher than the loss modulus G" across the entire frequency range ^[41]^. By analysing the trend of G′, it was possible to obtain information about the relative mechanical strength of the different systems and the crosslinking density within the PEI/BDDE network, which is directly proportional to the G′ value. Figure SI7A presents the frequency sweep curves for three different xerogels (i.e. SPEI1, SPEI2 and SPEI3), where the PEI:BDDE ratio increases from 1:1 for SPEI1 to 1:3 for SPEI3. It is evident that G′ decreased as the PEI:BDDE ratio increases, indicating a reduction in crosslinking density. This observation confirms our previous analysis: a lower BDDE content results in less crosslinking. Moreover, Figure SI7B shows the frequency sweeps of SPEI3, SPEI4 and SPEI5, where the PEI concentration was increased while maintaining a constant PEI:BDDE ratio (3:1). The observed increase in mechanical strength with increasing PEI content (reflected in the rise of the G′ value) can be attributed to the greater compactness and density of the system, as supported by the SEM micrographs.

| 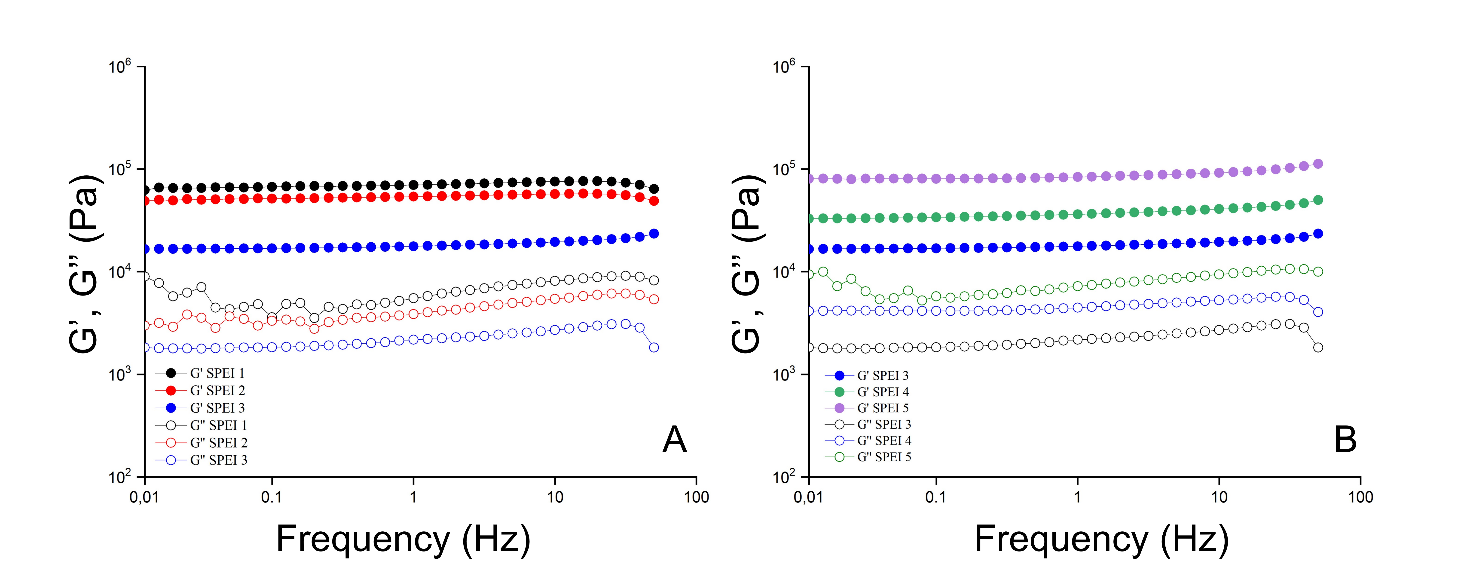 |
| --- |
| **Figure SI7.** Graphs associated with (A) the frequency sweep measurements of SPEI 1 (black), SPEI 2 (~~blue~~red), SPEI 3 (~~green~~blue) to evaluate the variation of G’ and G’’ with PEI:BDDE ratio; (B) the frequency sweep measurements of SPEI 3 (~~black~~blue), SPEI 4 (~~blue~~green), SPEI 5 (~~green~~purple), to examine the variation of G’ and G’’ with PEI concentration (w/w%). |

*Acetic acid Absorption tests.* Acetic acid absorption tests ^[33]^ were performed to evaluate the ability pf the various samples to absorb gaseous acetic acid. The presence of acetic acid whitin the xerogel was monitored by means of FTIR-ATR spectroscopy (Fig. 1A). The amount of absorbed acetic acid was determined through gravimetric analysis after the absorption test (AcOH_ab_%, Eq. 2, Tab. SI2).

**Table SI2.** Amount of total acetic acid absorbed by SPEI 1-5 after the absorption test (AcOH_ab_%) and the corresponding weakly (AcOH_wb_) and strongly (AcOH_sb_) bond acetic acid.

| \| Sample \| AcOH_ab_%  ° \| AcOH_wb_%  # \| AcOH_sb_%  ## \| \| --- \| --- \| --- \| --- \| \| PEI \| **111±2** \| **50±1** \| **61**±1 \| \| SPEI 1 \| 23±4 \| 16±1 \| 7±1 \| \| SPEI 2 \| 54±6 \| 37±3 \| 17±2 \| \| SPEI 3 \| 76±5 \| 42±3 \| 34±3 \| \| SPEI 4 \| 76±2 \| 44±4 \| 32±3 \| \| SPEI 5 \| 76±4 \| 49±2 \| 27±2 \|   °calculated with Eq. 2 #calculated with Eq. 3 ##calculated with Eq. 4 |
| --- | --- | --- | --- | --- | --- | --- | --- | --- | --- | --- | --- | --- | --- | --- | --- | --- | --- | --- | --- | --- | --- | --- | --- | --- | --- | --- | --- | --- |

From Tab. SI2, all the samples exhibited lower AcOH_ab_% and AcOH_sb_% (the fraction of strongly-bound acetic acid, Eq. 4), compared to pure PEI (AcOH_ab_: 111±2%, AcOH_sb_: 61±1% ). This behavior is probably due to the fact that part of the NH_2_ was involved in the epoxy bonds with BDDE, being unavailable for neutralizing acetic acid. When comparing the samples synthesized with varying the PEI:BDDE ratio, the amount of absorbed acetic acid (AcOH_ab_%) significantly increased with the PEI:BDDE ratio (23±4% for SPEI 1, 54±6% for SPEI 2, 76±5% for SPEI 3, Fig. SI8A, orange).

For samples made with different PEI concentrations while keeping the BDDE:PEI ratio constant, the absorbed acetic acid remained relatively unchanged (76±5% for SPEI 3, 76±2% for SPEI 4, 76±4% for SPEI 5, Fig. SI8B, orange).

| 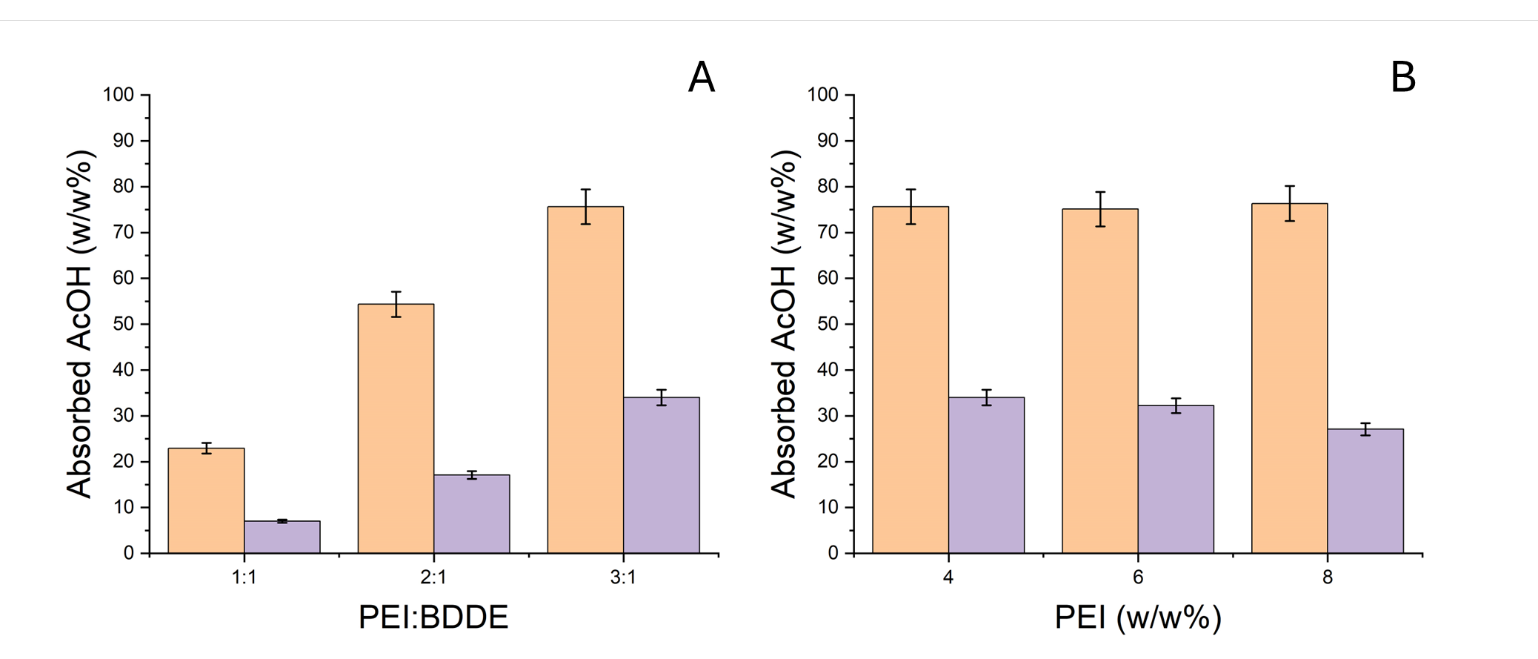 |
| --- |
| **Figure SI8.** Percentage of absorbed acetic acidafter the six days of absorption test (AcOH_ab_%, orange) and after the desorption test (AcOH_sb%,_ purple) for samples SPEI 1,2,3 by changing PEI:BDDE ratio (A) and SPEI 3,4,6 by changing PEI concentration (w/w%) (B). |

Desorption tests under low pressure were performed to explore the nature of the interaction between the xerogel and the acetic acid. By placing the xerogel under vacuum, a decrease in weight was observed until it reached a constant value after 36 h (Tab. SI2, AcOH_sb_% column). The achievement of constant weight, higher than the initial weight before the interaction with acetic acid, suggests the presence of residual acetic acid, likely remaining strongly bonded to the xerogel as ammonium acetate ^[33]^.

For the xerogels with varying PEI:BDDE ratios, the trend observed after the absorption test (AcOH_ab_) was similarly reflected in the desorption results (the AcOH_sb_, calculated with Eq. 3, is 7±1% for SPEI 1, 17±2% for SPEI 2, 34±3% for SPEI 3, Fig. SI8A, purple; the AcOH_wb_, calculated with Eq. 4, is 16±1% for SPEI 1, 37±3% for SPEI 2, 42±3% for SPEI 3). For the SPEI in which the variation in PEI concentration was examined while maintaining a constant PEI:BDDE ratio, no significant differences were observed, except for a slight decrease in AcOH_sb_ with increasing PEI concentration (AcOH_sb_ is 34±3% for SPEI 3, 32±3% for SPEI 4, 27±2% for SPEI 5, Fig. SI8B, purple; the AcOH_wb_ is 42±3% for SPEI 3, 44±4% for SPEI 4, 49±4% for SPEI 5).

To select the most promising system, we considered a balance between the capacity to absorb gaseous acetic acid and mechanical stability (as defined through rheology tests). The systems with a higher amount of free amino groups (SPEI 3,4,5, with a higher PEI:BDDE ratio) demonstrated a greater capacity to absorb both total acetic acid (AcOH_eq_) and AcOH_sb_. Although these three systems exhibited similar behaviors, SPEI 3 and 4 absorbed the highest amounts of AcOH_sb_% (34±3% and 32±2%, respectively). Furthermore, comparing SPEI 3 and 4, SPEI 4 showed a higher G′ value, indicating greater elasticity. On these bases, SPEI 4 was selected for further testing.


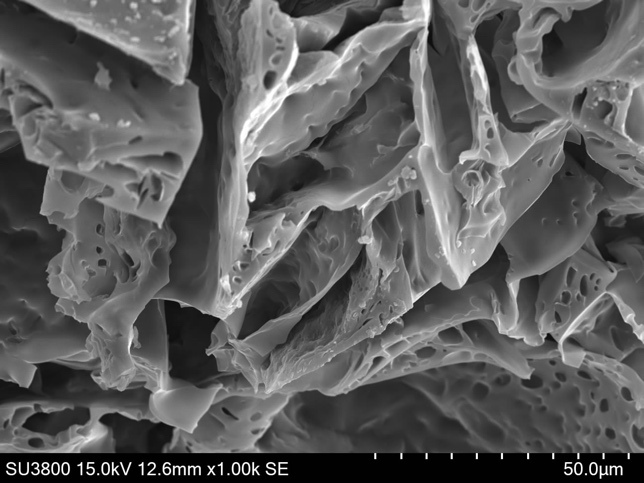


Figure SI9. SEM micrographs performed with 1.00kx magnitude of SPEI 4 after 3 cycles of absorption-desorption of acetic acid

In order to verify the physical and mechanical changes that the xerogel SPEI4 can undergo (or not) after the reuse of the material, a SEM analysis has been carried out after the third adsorption-desorption cycle (Figure 1A in the main text) and after the regeneration of the system by immersion in a 1M NaOH solution for 10 minutes ^[34]^ (Figure SI9). By comparing the image in Figure SI9 with the on reported in Figure SI5C (i.e. the SPEI4 before the first adsorption cycle) it is possible to observe that the morphology and the micro porosity of the system doesn’t change meaningfully. Moreover, a rheological analysis has been also carried out after the third regeneration. The results are shown in Figure SI10. It is evident that the rheological behaviour of the system after the third cycle of absorption/desorption (red symbols) is like the native SPEI4 (green symbols). Indeed, for the regenerated sample, the storage modulus G’ is still always higher than the loss modulus G” indicating that the system maintains a solid-like character typical of gels. The most important effect induced by the regeneration protocol is the growth of the G’ value attributable to an increase of the crosslinking density between the PEI chains constituting the network that doesn’t affect the absorption ability of the system (Figure 1A of the main text).


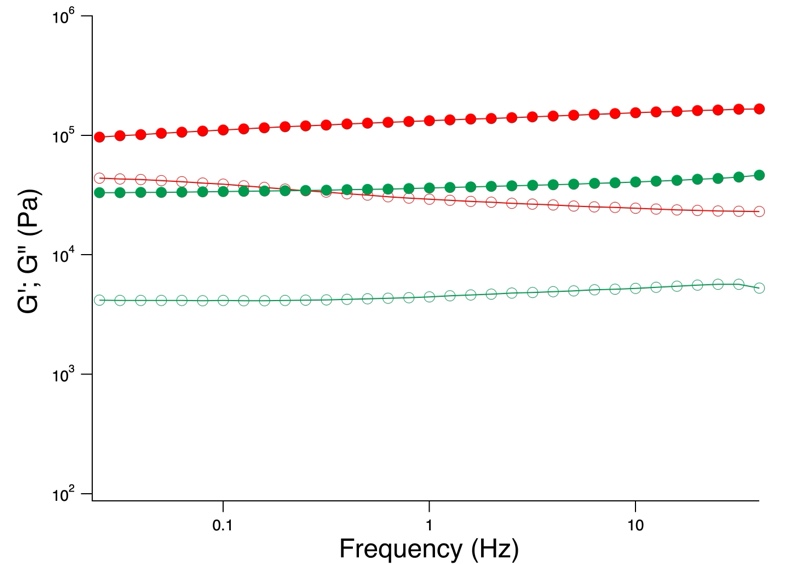


**Figure SI10.** Frequency sweep measurements of native SPEI 4 (green) and of SPEI 4 after the third regeneration (red). G’ corresponds to filled circles and G” with open circles.

References

[32] M. C. Fischer, A. Robb, *Topics Photogr Preserv* **1993**, *5*, 117–122.

[33] A. Zuliani, D. Bandelli, D. Chelazzi, R. Giorgi, P. Baglioni, *J Colloid Interface Sci* **2022**, *614*, 451–459.

[34] Q. Yang, T. Runge, *ACS Sustain Chem Eng* **2019**, *7*, 933–943.

[35] S. Demirci, N. Sahiner, *Ind Eng Chem Res* **2022**, *61*, 2771–2782.

[36] F. Zaaeri, M. Khoobi, M. Rouini, H. A. Javar, *International Journal of Polymeric Materials and Polymeric Biomaterials* **2018**, *67*, 967–977.

[37] A. M. Sajjan, H. G. Premakshi, M. Y. Kariduraganavar, *Journal of Industrial and Engineering Chemistry* **2015**, *25*, 151–161.

[38] M. L. Campbell, F. D. Guerra, J. Dhulekar, F. Alexis, D. C. Whitehead, *Chemistry - A European Journal* **2015**, *21*, 14834–14842.

[39] Y. Privar, I. Malakhova, A. Pestov, A. Fedorets, Y. Azarova, S. Schwarz, S. Bratskaya, *Chemical Engineering Journal* **2018**, *334*, 1392–1398.

[40] J. W. Goodwin, R. W. Hughes, *Rheology for Chemists: An Introduction*, The Royal Society Of Chemistry, Cambridge, **2001**.

[41] K. Almdal, J. Dyre, S. Hvidt, O. Kramer, *Polymer Gels and Networks* **1993**, *1*, 5–17.
